# Mesoporous Silica Nanoparticles for Targeting Subcellular Organelles

**DOI:** 10.3390/ijms21249696

**Published:** 2020-12-18

**Authors:** Miguel Gisbert-Garzarán, Daniel Lozano, María Vallet-Regí

**Affiliations:** 1 Departamento de Química en Ciencias Farmacéuticas, Instituto de Investigación Sanitaria Hospital 12 de Octubre i + 12, Universidad Complutense de Madrid, Plaza Ramón y Cajal s/n, 28040 Madrid, Spain; migisber@ucm.es (M.G.-G.); danlozan@ucm.es (D.L.); 2 Networking Research Center on Bioengineering, Biomaterials and Nanomedicine (CIBER-BBN), 28029 Madrid, Spain

**Keywords:** mesoporous silica nanoparticles, targeting, subcellular targeting, endosomal escape, mitochondria, nucleus, nanomedicine, stimuli-responsive, drug delivery

## Abstract

Current chemotherapy treatments lack great selectivity towards tumoral cells, which leads to nonspecific drug distribution and subsequent side effects. In this regard, the use of nanoparticles able to encapsulate and release therapeutic agents has attracted growing attention. In this sense, mesoporous silica nanoparticles (MSNs) have been widely employed as drug carriers owing to their exquisite physico-chemical properties. Because MSNs present a surface full of silanol groups, they can be easily functionalized to endow the nanoparticles with many different functionalities, including the introduction of moieties with affinity for the cell membrane or relevant compartments within the cell, thus increasing the efficacy of the treatments. This review manuscript will provide the state-of-the-art on MSNs functionalized for targeting subcellular compartments, focusing on the cytoplasm, the mitochondria, and the nucleus.

## 1. Introduction

Mesoporous silica materials have been widely studied since researchers from Waseda University [1] and the Mobil Oil Corporation [2] first reported them back in the early 90s. These bulk mesoporous materials offered (a) adjustable porous structures, (b) tunable and narrow pore size distributions (2–30 nm), (c) high pore volumes (ca. 1 cm^3^/g), (d) high specific surface areas (up to 1500 m^2^/g) and (e) high silanol density that allows further functionalization [3,4]. Given their excellent physico-chemical properties, mesoporous silica materials have been widely employed in many different fields, including catalysis [5,6], energy storage [7,8], or heavy metal adsorption [9,10], among others. 

In addition to those applications, the field of drug delivery has greatly benefitted from the use of this type of materials since Prof. Vallet-Regí and coworkers first reported their suitability to host and release therapeutic payloads in 2001 [11]. Given their excellent properties and promising biomedical features, scientists focused on translating such properties to the nanoscale dimension, leading to mesoporous silica-based nanoparticles (MSNs) resembling the physico-chemical characteristics found in the bulk materials and paving the way to multiple biomedical applications. Examples of these applications include bone tissue regeneration [12,13], antibacterial treatment [14,15], controlled drug delivery [16,17], gene transfection [18,19] or as templates for carbon-based biomaterials [20,21], among others. Aside from the above-mentioned biomedical applications, MSNs have been extensively employed as drug delivery carriers for cancer treatment. In this sense, research has focused on two main aspects namely, achieving on-demand drug delivery and addressing the nanoparticles specifically to cancer cells.

Because MSNs present an open porous structure, therapeutics can be easily loaded within the silica matrix. However, for the same reason, it is very easy for them to diffuse out of the pores, leading to nonspecific drug distribution and side effects. A smart approximation to overcome such drawback is the use of stimuli-responsive gatekeepers, which are molecular structures able to block the pore entrances to avoid drug leakage and open them only upon application of a specific stimulus at the tumor. The origin of the stimuli can be external (using specific equipment) or internal (due to up-/downregulated values of biomarkers). Examples of external stimuli are light, ultrasounds, or magnetic fields. Examples of internal stimuli include acid pH, overexpressed enzymes, or upregulated redox species. The study of stimuli-responsive mesoporous silica nanomaterials is beyond the scope of this review and the reader is encouraged to check out this information elsewhere [22,23,24].

In this review, we will focus on how MSNs can reach tumoral tissues and cancer cells, with emphasis on the specific targeting of subcellular organelles within target cells. 

## 2. Basic Principles of Cancer Nanomedicine

Ideally, nanoparticles should accumulate preferentially in the tumor, where they would exert their action without affecting healthy tissues. Nonetheless, the many different biological barriers that nanoparticles have to face upon administration may lead to reduced efficacy of the treatments and prevent the successful bench-to-bedside translation of nanomedicines [23,25]. Examples of such barriers include (a) fast bloodstream clearance, (b) lack of preferential accumulation in the tumor tissues, (c) lack of selective internalization in cancer cells, and (d) endosomal entrapment, among others. 

### 2.1. Targeting Tumor Tissues

The rationale for using nanoparticles (of any type) in cancer treatment relies on the Enhanced Permeability and Retention effect (EPR effect). It was first reported by Maeda and coworkers and, indeed, it is the basis of some commercialized nanomedicines [26]. According to Maeda et al., the EPR effect promotes the passive accumulation of nanoparticles in a tumor as a consequence of the poor lymphatic drainage, enhanced permeability, and the hypervasculature characteristic of many solid tumors [27].

Such passive accumulation of nanoparticles is greatly affected by features such as their size and surface chemistry. Regarding size, nanoparticles will avoid renal clearance if they are at least 10 nm in diameter, and will extravasate to the tumor if they are smaller than 400 nm. Nonetheless, this is still a controversial issue regarding the size that maximizes extravasation and subsequent cancer cell uptake. In this sense, some authors consider a size of ca. 300 nm as the most effective [28], while others propose a size of ca. 100 nm or below [29]. The surface chemistry directly affects the behavior of the nanoparticles in a biological fluid, as it determines the extent of plasma proteins adsorption onto the surface. These proteins form a protein corona that provides a biological entity to the particles, triggering their bloodstream clearance, and preventing tumor accumulation [30]. This phenomenon can be minimized by modifying the surface of MSNs with stealth moieties, such as polyethylene glycol or zwitterionic molecules, that prevent protein deposition through the creation of a hydration layer [23].

Even though the EPR effect constitutes a reliable strategy for accumulating nanomedicines in a tumor, its magnitude is greatly affected by the particularities of the tumor and the patient [31]. That non-universal passive accumulation has boosted the development of active strategies to enhance the delivery of nanocarriers. Examples of such strategies are particle-carrying tumor-tropic cells that spontaneously migrate to tumor tissues or nanoparticles functionalized with tumor-tropic peptides that promote their accumulation and tumor penetration [23].

### 2.2. Targeting Cancer Cells

Because cell membranes are negatively charged, the simplest approximation to trigger the cellular uptake of MSNs is engineering a cationic surface, which will lead to the internalization through electrostatic interactions [32]. Unfortunately, such a strategy does not provide any selectivity for cancer cells over normal cells. A widely employed approach for promoting the accumulation of the nanocarriers specifically in tumoral cells consists of functionalizing the surface with targeting ligands that selectively bind membrane receptors that are overexpressed only in the tumoral cells. 

There are several examples of antibody-functionalized nanoparticles. These structures show extremely high specificity for very specific membrane receptors. In this regard, MSNs have been modified with FDA-approved monoclonal antibodies such as Trastuzumab (HER2 receptor) [33,34] or Cetuximab (EGFR receptor) [35,36], among others, endowing them with great ability for targeting different types of tumoral cells. Similarly, aptamers show specificity comparable to that of antibodies, albeit being non-immunogenic and easy to produce. For instance, they can be employed to functionalize the surface of MSNs for targeting the EpCAM (EpCAM aptamer) [37,38,39] and MUC1 (MUC1 aptamer) [40,41] proteins or the nucleolin receptor (AS1411 aptamer) [42,43,44], among others.

Many authors have employed small commercially available molecules, such as folic acid, biotin, or boronic acids to target overexpressed vitamin receptors [45,46,47,48] or sialic acids [49], respectively. In addition, highly specific tumor targeting with MSNs can be achieved by synthesizing small molecules, such as benzylguanidine analogs that show high selectivity for the upregulated norepinephrine transporter of neuroblastoma cells [50]. In this sense, the development of peptide synthesis techniques has allowed for the functionalization of MSNs with many different peptidic molecules with affinity for very specific receptors. Examples of these peptides are RGD (αβ-integrins) [51,52], NGR (CD13 receptor) [53,54], NAPamide (melanocortin-1 receptor) [55] or IL-13 (IL-13R-α2) [56], among others. In addition to peptidic fragments, the use of biocompatible proteins has also yielded MSNs with high tumor specificity. In this regard, the overexpression of the transferrin receptor in many types of cancer has boosted the use of this protein as recognition moiety [57,58,59,60]. Additional approximations involve the use of proteins such as concanavalin-A (sialic acids) [61] or Aleuria Aurantia (sialyl-Lewis X antigen) [62], among others.

## 3. A Step Ahead: Subcellular Cancer Cell Targeting

### 3.1. Targeting the Cytoplasm: Endosomal Escape

Aside from accumulating in the tumor or being preferentially internalized by tumoral cells, nanoparticles should ideally be able to release their cargo properly within the cells. However, when nanoparticles are internalized through endocytosis they may end up sequestered within the acidic endosomes and lysosomes. This may have two main consequences: (1) the acidic environment might degrade the payload and (2) membrane-impermeable and/or poorly membrane-permeable therapeutics might not be able to exert the therapeutic action. There are two main approaches for inducing the disruption of the endo-lysosome, those internally triggered and those externally triggered. 

#### 3.1.1. Internally Triggered Endosomal Escape

Researchers mainly take advantage of the “proton sponge effect” to induce the endosomal escape via internal stimulus. For this disruptive effect to happen, nanoparticles should be functionalized with chemical moieties that show buffering capacity and the pH of such vesicles, which would lead to the basification of the endo-lysosomes. The cell would influx chloride ions along with water molecules to counteract that proton removal. Finally, the endo-lysosomes would swell owing to the large amount of water molecules, which would finally lead to the disruption of the vesicles and subsequent nanoparticle release [63]. 

Weiss et al. developed MSNs functionalized with poly(amidoamine) dendrimers and further modified with folic acid, triggering their selective uptake in KB cells [64]. Then, the large number of amino groups in the dendrimer induced the endosomal escape into the cytoplasm via proton sponge effect, where the overexpressed glutathione triggered the release of a redox-responsive derivative of colchicine from the mesopores. Chen et al. recently reported metal-phenolic networks consisting of tannic acid complexed with Fe^III^ ions as coatings for MSNs [65]. At pH > 7, Fe^III^ ions were complexed to three tannic acid molecules, forming a tris complex. However, the acid pH of the endo-lysosomes induced a tris-to-bis complex transition as a consequence of the protonation of the phenolic molecules of tannic acid, which finally led to the endosomal escape of the particles in MDA-MB-231 cancer cells. 

**Figure 1:**
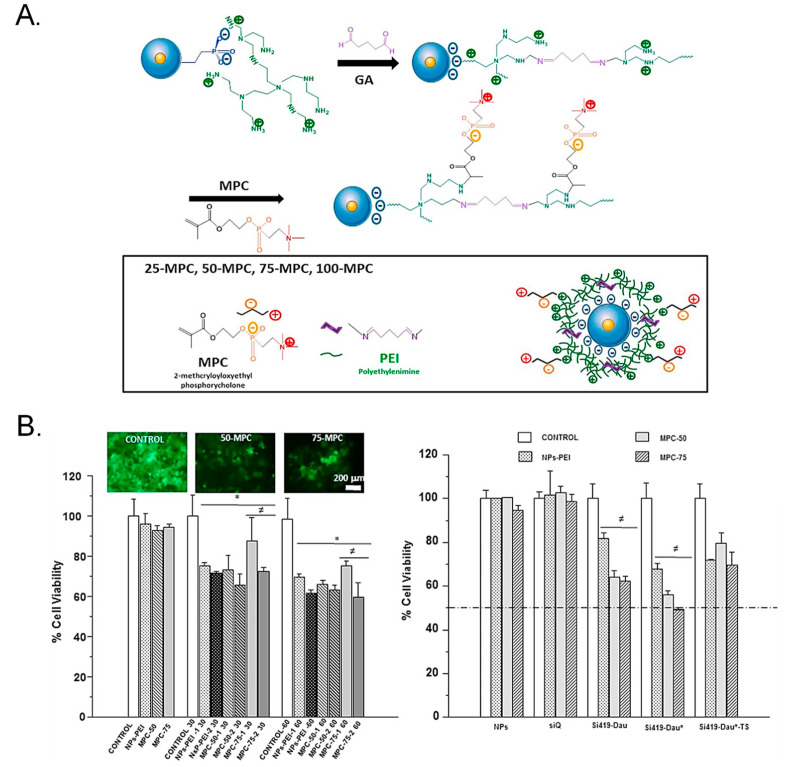
(**A**) Schematic representation of the mesoporous silica nanoparticles (MSNs). MSNs were functionalized with PEI for siRNA loading and further modified with a phosphorylcholine derivative to introduce zwitterionic groups to endow the MSNs with antifouling features. (**B**) Cytotoxic effect of the functionalized MSNs. The highest cytotoxicity was observed for the groups containing the siRNA and the drug daunorubicin. Reproduced from Ref. [67] with permission from Elsevier.

Polyethyleneimine (PEI) is a cationic polymer that finds broad application for the transfection of nucleic acids, which need to be released in the cytoplasm to exert their therapeutic action. A major drawback of chemotherapy is drug resistance, which renders the cytotoxic less effective. In this sense, a smart approach consists of transfecting a gene capable of inhibiting the drug-resistant character of cancer cells and subsequently delivering a cytotoxic to the sensitized cells. In this regard, Chen et al. functionalized the surface of MSNs with PEI to incorporate a short hairpin RNA (shRNA) for silencing ABCG2 protein [66]. PEI successfully triggered the escape and induced the release of doxorubicin and the shRNA. As a consequence, the drug-resistant protein ABCG2 was no longer produced, diminishing the amount of doxorubicin excreted by the cancer stem cells and inhibiting their proliferation both in vitro and in vivo. TWIST is a protein that is linked to drug resistance and cancer progression. Sánchez-Salcedo et al. reported a PEI-coated Fe_3_O_4_@SiO_2_ core-shell nanocarrier with reduced protein adsorption features for the knockdown of TWIST [67]. Upon accomplishment of the PEI-mediated endosomal escape through proton sponge effect, an anti-TWIST small interfering RNA (siRNA) and the chemotherapeutic daunorubicin were released. Then, the siRNA increased the sensitivity to the drug, resulting in enhanced cytotoxicity to ovarian cancer cells OVCAR8 (Figure 1).

The endosomal escape can also be accomplished by using amino acids with buffering capacity. In this sense, the imidazole ring of histidine has a pKa of ca. 6 [68] and shows buffering capacity at the pH of the endocytic pathway. Bilalis et al. reported the functionalization of the surface of MSNs with poly(L-histidine) using a surface-initiated ring-opening polymerization [69]. They showed that the peptidic coating could act as pH-responsive gatekeeper but they did not verify the endosomal escape features of the system. Nonetheless, a dramatic increase in the zeta potential was observed at pH 5 (that of the lysosomes), compared to that at pH 7.4, demonstrating the buffering capacity of the system and its usefulness as endosomal escape agent. Similarly, Li et al. took advantage of just the imidazole motif to design dendritic MSNs for co-delivery of doxorubicin and survivin shRNA-expressing plasmid [70]. The surface of the particles was functionalized with imidazole to accomplish endosomal escape and the shRNA was electrostatically loaded on the surface. The release of the shRNA in the cytoplasm led to the inhibition of survivin, increasing the sensitization of QGY-7703 cells to the co-delivered doxorubicin in vitro and in vivo. 

#### 3.1.2. Externally Triggered Endosomal Escape

Aside from taking advantage of the acid pH of the endo-lysosomes, it is possible to induce the escape of the particles by functionalizing the surface of the MSNs with chemical moieties able to generate reactive oxygen species (ROS) upon light irradiation. The generation of ROS induces the peroxidation of the lipid bilayer of the endo-lysosomes, altering its fluidity and permeability and triggering its destabilization and subsequent nanoparticle escape [71]. 

The simplest approximation consists of loading a photosensitizer within the mesoporous silica matrix. Hai et al. designed DNA-capped MSNs loaded with indocyanine green for chemo-photothermal therapy [72]. Indocyanine green is a photothermal and photosensitizing agent that generates heat and ROS upon irradiation with near-infrared light (780 nm). Hence, the nanoparticles first internalized in HeLa cells and were then irradiated with the laser, leading to the generation of ROS that triggered the destabilization of the endosomes. In addition, the heat generated by the photosensitizer induced the dehybridation of the DNA strands, triggering the doxorubicin release in the cytoplasm. The photosensitizer can also be grafted on the surface. Niedermayer et al. reported pH-responsive, folic acid-targeted MSNs to which the photosensitizer AlPcS_2a_ was grafted [73]. The nanocarrier was efficiently internalized in KB cells thanks to the overexpressed folate receptors and then the acid pH triggered the release within the endo-lysosomes. After that, the particles were irradiated with red light (639 nm), generating ROS and inducing the endosomal escape that allowed for the release of a membrane-impermeable compound in the cytoplasm. 

**Figure 2:**
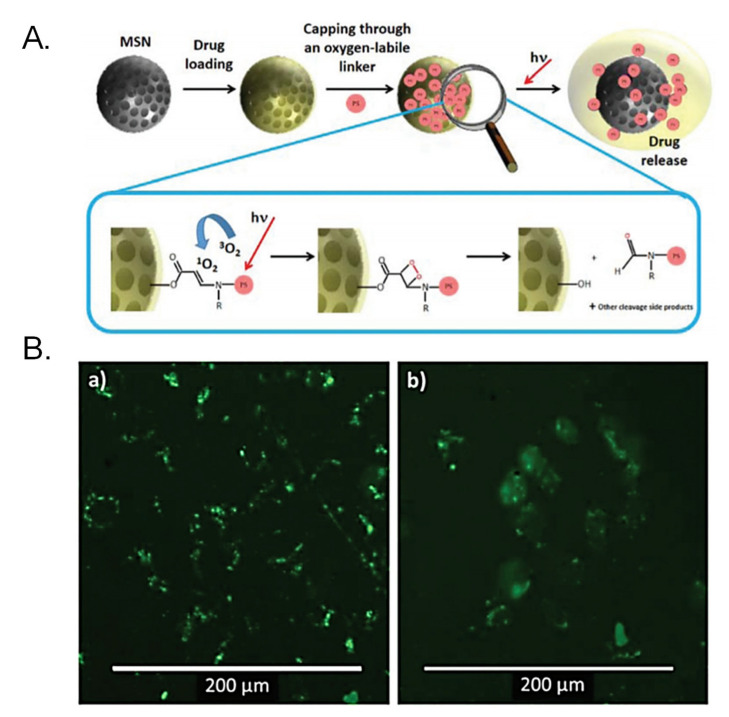
(**A**) Schematic representation of MSNs engineered for achieving endosomal escape through reactive oxygen species (ROS) generation. A visible light-sensitive porphyrin is grafted to the surface through a ROS-responsive linker that is cleaved when upon light irradiation, triggering endosomal escape and drug release. (**B**) Calcein assay for assessing endosomal escape: Calcein is a membrane-impermeable dye that is unable to cross the lipid bilayer of the endo-lysosomes in the dark. When light is applied, ROS generated destabilizes the membrane and calcein diffuses into the cytoplasm. Reproduced from Ref. [75] with permission from The Royal Society of Chemistry.

The generation of ROS can also be employed for cleaving ROS-responsive bonds, such as those based on aminoacrylates. Vijayakameswara Rao et al. engineered a gatekeeper for MSNs based on a Gd-DOTA complex grafted to the surface through an aminoacrylate-based bond [74]. The surface was further functionalized with a polyethylene glycol containing the photosensitizer chlorin e6 and the particles were incubated with SCC-7 cells. Then, upon application of 660-nm light, the photosensitizer induced the generation of ^1^O_2_ that triggered the cleavage of the stimuli-responsive bond as well as the endosomal escape, leading to enhanced drug release and cytotoxic effect both in vitro and in vivo. Martínez-Carmona et al. reported the use of photosensitizers as both ROS generators and gatekeepers for MSNs [75]. A porphyrin able to generate ^1^O_2_ when irradiated with visible light was grafted to the particles via an aminoacrylate-based bond, sealing the pore entrances in the dark. The particles were readily internalized in human osteosarcoma cells where they localized in the lysosomes. Then, the application of visible light induced the generation of ROS that triggered the destabilization of the vesicle and the cleavage of the stimuli-responsive linker, thereby leading to the release of the cargo in the cytoplasm of the cells (Figure 2). 

### 3.2. Mitochondrial Targeting

One of the main drawbacks of current antitumoral drugs, such as doxorubicin or topotecan, is the development of drug resistance, which renders the drugs useless and leads to the failure of the treatments. Given that each chemotherapeutic exerts its action on a particular organelle, delivering the drugs near such a particular organelle may help to avoid drug resistance. In this sense, researchers have paid much attention to mitochondrial targeting, as mitochondria are involved in cell apoptosis, cell metabolism, and ROS generation [63,76]. 

#### 3.2.1. Triphenylphosphine Derivatives to Target Mitochondria

Because the mitochondrial membrane is negatively charged, the use of positively charged substances seems appealing for accumulating the cytotoxics within the mitochondrial matrix. In this sense, current approximations for improving mitochondrial internalization include linking a lipophilic cation (alkyltriphenylphosphonium and others) to a bioactive compound. Among them, Triphenylphosphine derivatives (TPP) are one of the most promising and widely used mitochondria-targeting ligands, especially in combination with MSNs [63,76]. 

**Figure 3:**
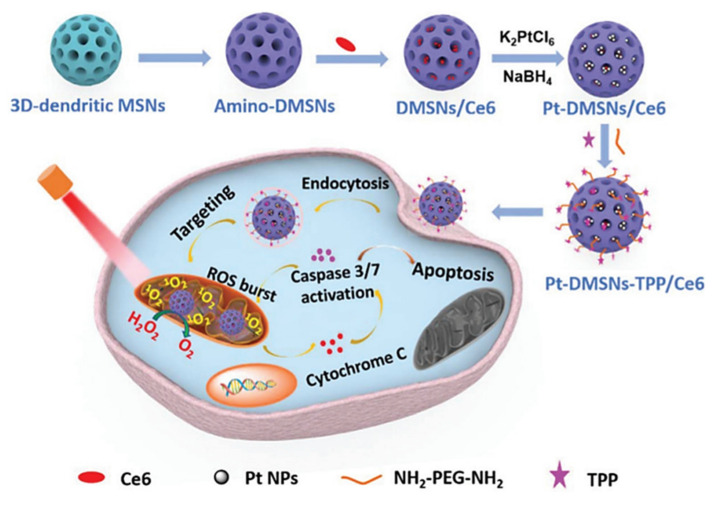
Schematic representation of mitochondria-targeted dendritic MSNs for photodynamic therapy. MSNs were functionalized with triphenylphosphonium (TPP) for mitochondrial targeting and loaded with the photosensitizer Ce6. Then, Pt nanoparticles showing peroxidase-like nanozyme activity were attached to the surface to catalyze the conversion of intracellular H_2_O_2_ into oxygen in the tumoral cells. Finally, Ce6 generated ROS upon light irradiation, leading to synergistic cell death. Reproduced from Ref. [78] with permission from The Royal Society of Chemistry.

Qu Q et al. reported TPP-functionalized MSNs (MSNP-PPh3) loaded with doxorubicin [77]. Co-localization of mitochondria and MSNs was demonstrated in HeLa tumoral cells by fluorescence analysis, showing the targeting efficacy. The authors also demonstrated the effective release of doxorubicin to mitochondria by MSNP-PPh3 and a successful escape from lysosomes. The DOX-loaded nanomaterial led to reduced cellular adenosine triphosphate (ATP) production and mitochondria membrane potential, indicating mitochondria dysfunction and resulting in a significant reduction in HeLa cell viability. Cai X et al. reported a new class of mitochondria-targeted three-dimensional (3D) dendritic MSN nanospheres for the delivery of high concentrations of the hydrophobic photosensitizer chlorin e6 (Ce6) in A549 lung cancer cells (Figure 3) [78]. Photodynamic therapy (PDT) in cancer presents some limitations, such as short lifetime or tumor hypoxia. To address the latter, the authors synthesized peroxidase-like nanozyme Pt NPs with ability to catalyze the conversion of intracellular H_2_O_2_ into oxygen in the tumoral cells. Then, MSNs were modified with the Pt NPs and further functionalized with TPP for mitochondrial targeting (Pt-DMSNs-TPP/Ce6). This nanocarrier induced an intracellular ROS burst in A549 cells due to the mitochondrial targeting capacity, resulting in cell apoptosis. Viability studies exhibited 80% of tumoral cell death induced by Pt-DMSN-TPP/Ce6 nanosystem upon light irradiation at 660 nm, eradicating the hypoxia problem. An alternative strategy was developed by Cheng R et al. [79] to clarify the mechanism of selenite (Na_2_SeO_3_)-mediated cancer cell death. For that purpose, the authors designed a mitochondria-targeted nanoprobe (Mito-N-D-MSN) able to monitor the changes in mitochondrial hydrogen selenide (H_2_Se) and superoxide anion (O_2_^·-^) changes simultaneously in HepG2 tumoral cells. This MSN-based nanodevice was loaded with two fluorescent nanoprobes and further modified with TPP. The in vitro results indicated that the mitochondrial H_2_Se content gradually increased, while the O_2_^·-^ content remained unaffected in HepG2 cells under hypoxic environments, suggesting that the antitumoral mechanism of Na_2_SeO_3_ comprises non-oxidative stress in the real tumor microenvironment. 

Aside from endowing the MSNs with mitochondrial targeting, they can be modified to achieve sequential cell-to-organelle recognition. In this regard, Sun K et al. developed an iron oxide@SiO_2_ core-shell nanosystem functionalized with folate and TPP for membrane and subsequent mitochondrial targeting (Fe_3_O_4_@MSN-TPP/PEG-FA) for cancer therapy [80]. The nanocarrier was loaded with doxorubicin and the catalase inhibitor 3-amino-1,2,4-triazole (AT). Doxorubicin triggered nicotinamide adenine dinucleotide phosphate oxidases (NOXs) activation, which induced the formation of H_2_O_2_. Catalase inhibition led to the preservation of H_2_O_2_, forming hydroxyl radicals in the presence of the iron ions nearby mitochondria and exerting significant death on MGC-803 and MCF-7 cells. 

A similar approximation was reported by Wang L et al., who developed TPP-functionalized MSNs coated with a folic-acid targeted, pH-controlled lipid bilayer [81]. The particles were loaded with 2,2’-azobis[2-(2-imidazolin-2-yl) propane] dihydrochloride (AIPH) and the liposomes were loaded with docetaxel, forming the final core-shell system by self-assembly throughout the liposomes hydration (AIPH/MSN-TPP@Lipo/DTX-FA). AIPH/MSN-TPP@Lipo/DTX-FA nanosystem successfully internalized in breast tumoral cells through FA receptor-mediated endocytosis. The liposomes were destabilized by the acid pH of the lysosomes and docetaxel was released. Then, the AIPH/MSN-TPP nanoparticles escaped the lysosomes and targeted mitochondria, releasing AIPH and inducing temperature-activated alkyl radicals burst (oxidative damage), showing synergistic effect both in vitro and in vivo. The selective recognition of tumoral cells can also be accomplished using hyaluronic acid to target overexpressed CD44 receptors. In this sense, Naz S et al. reported enzyme-responsive MSNs bearing TPP on the surface and further functionalized with hyaluronic acid (HA) as membrane targeting and pore capping agent [82]. HA triggered the internalization in CD44 receptor-positive cells and predominantly accumulated in mitochondria thanks to TPP. Then, HAase, which is overexpressed in cancer cells, induced the degradation of HA and triggered DOX release in MGC-803 tumoral cells, leading to significant cell death.

#### 3.2.2. Other molecular Agents to Target Mitochondria

**Figure 4:**
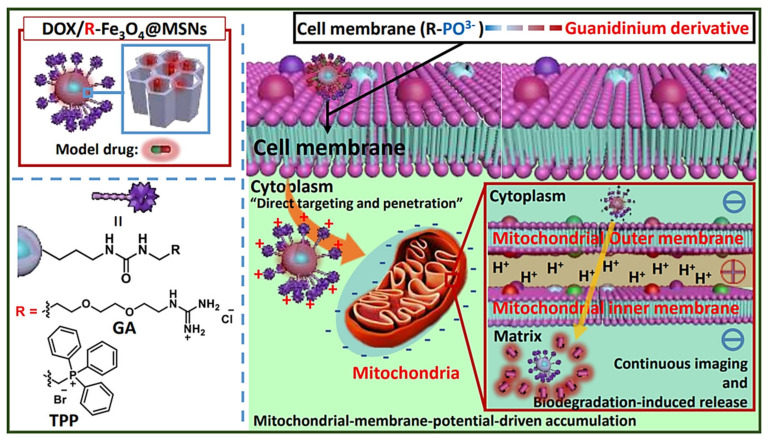
Schematic representation of MSNs functionalized with guanidinium derivatives for mitochondrial targeting. The GA-functionalized MSNs accumulated faster into the mitochondria, compared with the TPP derivatives. Reproduced from Ref. [83] with permission from The Royal Society of Chemistry.

Even though most of the systems are based on using TPP for targeting the mitochondria, there are some other approximations. For instance, Ahn J et al. proposed the use of guanidinium derivatives (GA) as mitochondrial targeting agents (Figure 4) [83]. They designed GA-targeted, DOX-loaded Fe_3_O_4_@SiO_2_ nanoparticles (DOX/GA-Fe_3_O_4_@MSNs) The nanosystem showed competent mitochondria-targeting capability in a co-localization, microscopic, and fluorometric analysis. DOX/GA-Fe_3_O_4_@MSNs induced a significant decrease in HeLa tumoral cell viability while the GA-Fe_3_O_4_@MSNs did not induce any change. Remarkably, GA induced higher mitochondrial targeting than that observed for a control group of such nanoparticles functionalized with TPP, highlighting the high affinity of GA. Another approach was reported by Liu J et al., who developed mitochondria-targeted, silica-coated gold nanorods (AuNR@MSN) by functionalizing the surface with the mitochondria-targeting peptide RLA ([RLARLAR]_2_) [84]. The hybrid was loaded with indocyanine green for dual photodynamic and photothermal therapy and the pores were capped with β-cyclodextrin and further functionalized with RLA. In addition, a charge-reversible polymer was hosted to provide stealth property. In this context, the weak acidity in the tumor tissue could produce the dissociation of the charge-reversible polymer, exposing the RLA peptide for cell internalization and subsequent mitochondria accumulation in MCF-7 tumor cells. After 808 nm NIR light irradiation, AuNR@MSN-ICG-RLA/CS (DMA)-PEG induced tumor cell apoptosis in vitro because of ROS generation and local hyperthermia via mitochondria. These results were confirmed in vivo where this nanodevice showed antitumoral effect with minimal side effect, after specific accumulation at tumor location, improving combination therapy of PDT and PTT. 

#### 3.2.3. Compounds Acting on Mitochondria

Hu J et al. [85] proposed a new approach for enhanced tumor chemotherapy based on ROS-triggered self-accelerating drug release MSNs (T/D@RSMSNs). The particles were loaded with DOX and α-tocopheryl succinate (α-TOS) and gated by PEGylated β-cyclodextrins through a ROS-cleavable thioketal linker. α-TOS is a vitamin E analog that acts as a ROS mediator after interacting with the mitochondrial respiratory complex II. T/D@RSMSNs released doxorubicin and α-TOS and induced an increase in intracellular ROS concentration in human breast cancer (MCF-7) cells, triggering the cleavage of the thioketal linker TK linkage for further doxorubicin release, both in tumoral MCF-7 cells in vitro studies and in BALB/c nude mice tumor xenograft models, decreasing tumor weight and volume, compared with others similar nanosystems based on ROS-responsive strategies.

Aside from conventional chemotherapeutics, researchers have paid attention to the encapsulation of less typical drugs with antitumoral activity. In this regard, Choi et al. developed PEGylated polyaminoacid-capped MSNs (CMSN-PEG) for celastrol delivery to cancer cells [86]. Celastrol is extracted from the traditional Chinese medicinal plant Tripterygium wilfordii and several studies have shown antitumoral effects in vitro and in vivo by targeting mitochondria. The celastrol-loaded nanosystem showed controlled drug release and induced apoptosis of SCC-7, SH-SY5Y, and BT-474 tumoral cell lines in vitro, related to decreased HIF-1α protein levels and other proteins implicated in mitochondrial apoptosis pathway. In vivo, this novel nanosystem significantly decreased solid tumor presence in SCC7 tumor-bearing xenograft model, inducing a rise in apoptotic markers and stimulating the reduction of CD31 and Ki67 proliferation markers. An alternative tumor tissue-specific drug is Umbelliferone, a natural coumarin derivative. Kundu M et al. [87] designed umbelliferone-loaded MSNs capped with pH-sensitive poly acrylic acid (PAA) and further functionalized with folic acid (Umbe@MSN-PAA-FA). Umbe@MSN-PAA-FA nanosystem induced a decrease in cell viability due to both oxidative stress and mitochondrial damage in MCF-7 cells, compared with free Umbelliferone. This nanohybrid displayed discretely drug loading capability, enhanced tumoral cell targeting, and effective pH-controlled Umbelliferone release properties. Thanks to the optimal drug bioavailability in the tumoral mass, Umbe@MSN-PAA-FA nanocarrier showed a significant decrease in tumor size on an in vivo solid tumor model in Swiss albino mice, compared with free umbelliferone, without negative effects in other vital organs. 

Luo GF et al. [88] attached a TPP-modified antibiotic peptide (KLAKLAK)_2_ to the surface of topotecan-loaded MSNs via disulfide bonds. Then, a charge reversal polyanion poly(ethylene glycol)-blocked-2,3-dimethylmaleic anhydride-modified poly(L-lysine) (PEG-PLL(DMA)) was added to the surface via electrostatic interactions. In this manner, the DMA block degraded in the acidic tumor microenvironment, removing the outer shielding layer and triggering the uptake in increasing ubiquitous KERATIN- forming tumor cell line HeLa. Finally, the TPP-modified peptide was released from nanodevice by the overexpressed glutathione, targeting the mitochondria and showing synergistic specific cancer cell death.

An alternative new antibacterial peptide delivery strategy was designed by Cao J et al. as a cancer treatment approach [89]. Given that many antibacterial peptides lead to in hemolysis in vivo, Cao J et al. synthesized a novel antibacterial peptide RGD-hylin a1 with reduced hemolysis and loaded it within the pores of the MSNs (RGD-hylin a1-MSN). This nanocarrier released RGD-hylin a1 in a pH-controlled manner at acid pH, inducing the reduction of mitochondrial membrane potential and leading to cancer cell death, and non-effect in pH = 7. In vivo, this nanocarrier reduced (50–60%) solid tumor volume and weight after intravenous administration in tumor-bearing mice at low dosage via intravenous injection compared with control non-treated group, without inducing toxicity. 

As shown in the review, MSNs have been widely engineered for targeting the mitochondria with extraordinary efficacy, increasing cancer cell death and decreasing tumor growth and size in vitro and in vivo. Nonetheless, it should be mentioned that these nanosystems also find application in other diseases, such as Alzheimer’s disease (AD). In this sense, several AD therapies have focused on amyloid-β targeted treatments, but recent studies have demonstrated that tau pathway is connected with clinical expansion of AD signs, indicating that can be a possible therapeutic target. In this regard, Chen Q et al. loaded the MSNs with the tau aggregation inhibitor methylene blue (MB) and further functionalized them with a hyperphosphorylated tau binding agent (Amino-T807) [90] as well as ultrasmall ceria nanocrystals (CeNCs) and iron oxide nanocrystals (IONCs). CeNCs exhibited high ROS activity and antioxidant properties in mitochondrial oxidative-stress-induced damage therapy in Alzheimer’s disease, acting as tau hyperphosphorylation inhibitors. Both in vitro and in vivo results showed that this complex nanocarrier decreased Alzheimer’s disease consequences, diminishing mitochondrial oxidative stress and inhibiting tau hyperphosphorylation and neuronal apoptosis, yielding a synergistically improved therapeutic alternative.

### 3.3. Nuclear Targeting

As previously mentioned, the problems that chemotherapeutics have to face are drug resistance and subsequent cell exocytosis. In this sense, drug-loaded nanocarriers can also be functionalized for targeting the nucleus, delivering their cargo nearby their target [76,91]. It is well-known that there are numerous pore complexes of 20-70 nm in diameter on the nuclear membrane, which are the specific passages through which substances of up to 40-60 kDa diffuse from and to the cytoplasm. Given that the passive diffusion through the nuclear envelope is restricted by the size of those complexes, nanoparticles need a nuclear localization signal or sequence (NLS), which are peptides for recognizing specific transport receptors to initiate the trans-nuclear membrane penetration process [92]. The internalization into the nucleus is mediated by importins. First, NLS peptides bind importin α and, then, importin β binds them to form a complex that finally transports the target compound into the nucleus [93]. 

**Figure 5:**
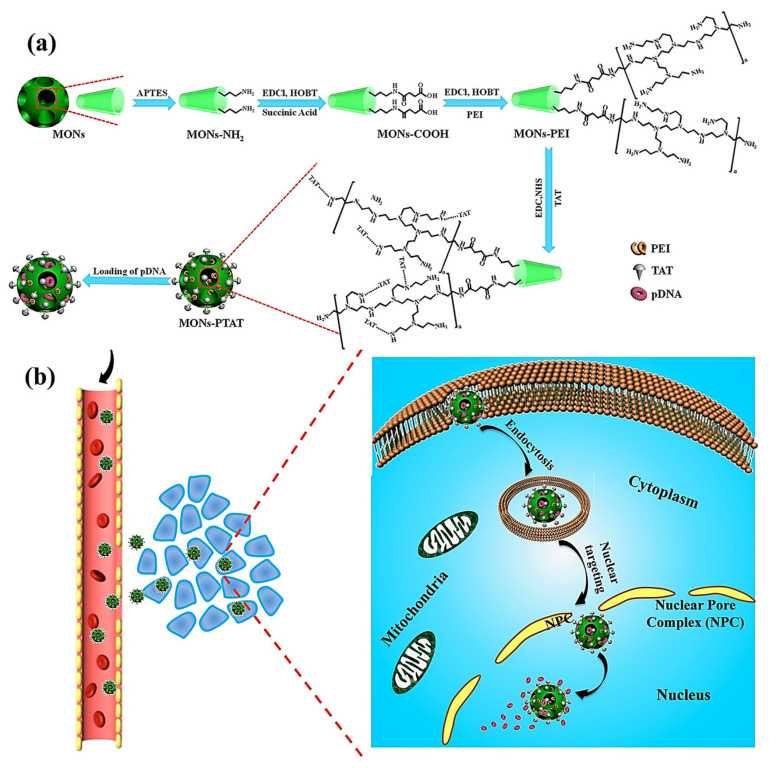
(**a**) Schematic representation of TAT-targeted MSNs for nucleic acid transfection. The plasmid was loaded into the PEI-TAT mesh, leading to enhanced nuclear accumulation and encoding green florescence protein through a model plasmid. (**b**) Schematic representation of the mode of action of the nanocarrier. Reproduced from Ref. [94] with permission from Wiley.

Wu M et al. reported the synthesis of PEI-coated, TAT-targeted MSNs for nuclear transfection of a model plasmid that encoded green fluorescent protein [94]. They demonstrated that the combination of PEI + TAT provided the highest plasmid loading capacity, probably due to the highly positive surface, and protected the nucleic acids from degradation. Finally, they demonstrated the nuclear targeting and efficiently encoded the green protein without affecting the viability of the cells (Figure 5).

The most extended strategy involves the design of dual-targeted nanoparticles bearing a membrane and a nuclear targeting for sequential cell-to-organelle recognition. In this regard, Zhao J et al. reported MSNs whose surface was functionalized with a fluorescent derivative of the HIV-1 transactivator (NLS) peptide (TAT-FITC), for nuclear targeting, and the peptide YSA-BHQ1 for binding the EphA2 membrane receptor [93]. In addition, citraconic anhydride was used to invert the charge of the TAT peptide in neutral or weak alkaline conditions so that the positively charged YSA peptide could combine with TAT through electrostatic attraction, avoiding nonspecific TAT interactions with normal cells. The nanocarrier was successfully internalized by MCF-7 tumoral cells via YSA recognition. Then, the acid pH of the lysosomes cleaved the citraconic anhydride and triggered the lysosomal escape and subsequent nuclear translocation, where DOX was rapidly released, inducing an apoptotic effect in the cells. Wu Z et al. synthesized dual-targeted, enzyme-responsive MSNs for anticancer therapy and imaging [95]. They doped the MSNs during the synthesis with Eu^3+^ and Gd^3+^ for carrying out bioimaging. Then, TAT was attached to the surface and the particles were further coated with HA to mask the peptide as well as to provide membrane targeting and gatekeeping. The in vitro evaluation showed that HA degraded upon a lysosome HA decomposition by HAase, inducing the exposition of nuclear target TAT, releasing camptothecin into the nucleus and stimulating tumoral cell death.

Murugan C et al. developed topotecan-loaded MSNs functionalized with the TAT peptide, to which RGD-functionalized poly(acrylic acid) and citraconic anhydride-metformin were added [96]. The latter components acted as gatekeepers and as masking agents of the positively charged TAT. The nanosystem was effectively internalized by MDA-MB-231 tumoral cells via integrin targeting. The lysosomal pH triggered metformin drug release and exposed again the TAT peptide, achieving endosomal escape and nuclear translocation. Then, topotecan was successfully released into the nucleus, leading to synergistic antitumoral effect and reducing tumor growth without significant toxicity associated with mice.

Aside from using NLS peptides, the nucleus can be targeted by taking advantage of the properties of gold nanoclusters, which show unique nuclei staining properties. In this sense, Croissant et al. [97] reported the synthesis of gemcitabine/doxorubicin-loaded MSNs functionalized with BSA-coated gold nanoclusters (MSN-AuNC@BSA) acting as gatekeepers via electrostatic interactions. Drug release took place at acid pH owing to the disruption of such interactions, triggering HeLa tumoral cell death. In addition, the BSA-coated nanoclusters were released at acid pH as well, being able to stain the nuclei for cancer cell imaging. The authors showed a robust specific fluorescence of the nanocarrier in the tumors induced in mice, indicating the high value of this type nanotheranostic platform based on MSNs and gold-protein clusters in cancer therapy and diagnosis. 

The different nanosystems able to target mitochondria or nucleus are summarized in Table 1:

## 4. Future Perspectives and Conclusions

The great physico-chemical and biocompatible features that MSNs exhibit has fueled the design of many different nanosystems specially designed for the treatment of various diseases. Yet it is true that no silica-based nanocarrier has reached the clinic so far, the US Food and Drug Administration (FDA) recognizes silica as safe. It is usually employed as excipient in drug formulations and as a dietary supplement [98,99]. Indeed, there are already examples of studies on silica materials conducted or being conducted in humans, highlighting the promising future that this kind of materials may have and the interest that they arouse in clinicians. 

Phillips et al. reported a human trial where they evaluated the usefulness of small 7-nm silica nanoparticles (c-dots) for imaging purposes in patients suffering metastatic melanoma [100]. The c-dots were functionalized with the cyclic peptide cRGDY for targeting endothelial cells involved in angiogenesis, observing that the silica nanoparticles could target the tumor while being well tolerated by the patients. In view of such promising results, the Memorial Sloan Kettering Cancer Center is currently recruiting candidates to evaluate whether c-dots can be employed for detecting lymph nodes during cancer surgery (ClinicalTrials.gov Identifier: NCT02106598) and imaging brain tumors (ClinicalTrials.gov Identifier: NCT03465618).

Regarding mesoporous silica materials, Bukara et al. reported the first study of ordered mesoporous materials in men [101]. They formulated poorly soluble fenofibrate in 500-nm mesoporous silica, which was orally administered to healthy men (average age: 46.4), demonstrating that the mesoporous silica materials were well-tolerated and safe. In addition, they showed that higher drug absorption was achieved compared to a marketed product. Furthermore, the Karolinska Institutet (Sweden) has recently submitted to the FDA the results (pending evaluation) of a human trial evaluating the safety profile of doses of up to 9 g/day of mesoporous silica microparticles as a food additive (ClinicalTrials.gov Identifier: NCT03667430).

It has been made clear throughout this review that incorporating subcellular targeting moieties may increase the efficacy of nanoparticle-based cancer treatments because drugs can be delivered closer to their target (mitochondria, nucleus…). Hence, given that there is real interest in translating this kind of nanomaterials into the clinic, the next step should be the design of clinical trials involving (1) mesoporous silica nanoparticles, so their promising features would be finally verified and (2) simple but effective structures for increasing the accumulation of the particles in the tumors along with the subcellular targeting or relevant organelles. 

## Figures and Tables

**Table 1:** Different approximations for the design of organelle-targeted MSNs.

Carrier Type	Organelle	Organelle Targeting Molecule	Drug	Cell Line(s)	In Vivo	Application	Ref
MSNs	Mitochondria	TPP	DOX	HeLa	X	Cancer	[77]
DMSNs		TPP	X	A549	X	Cancer	[78]
MSNs		TPP	X	HepG2	X	Cancer	[79]
Fe_3_O_4_@MSN		TPP	DOX	MGC-803, MCF-7	X	Cancer	[80]
MSNs		TPP	AIPH and DTX	MDA-MB-231	Mice	Cancer	[81]
MSNs		TPP	DOX	MGC-803	X	Cancer	[82]
Fe_3_O_4_@MSN		Guanidinium	DOX	HeLa	X	Cancer	[83]
AuNR@MSN		(RLARLAR)_2_	X	MCF-7	Mice	Cancer	[84]
MSNs		α-tocopheryl succinate	DOX	MCF-7	Mice	Cancer	[85]
MSNs		Celastrol	Celastrol	SCC-7,SH-SY5Y, BT-474	Mice	Cancer	[86]
MSNs		PAA	Umbelliferone	MCF-7	Mice	Cancer	[87]
MSNs		TPP, (KLAKLAK)_2_	Topotecan	HeLa	X	Cancer	[88]
MSNs		RGD-hylin a1	RGD-hylin a1	HeLa	Mice	Cancer	[89]
MSNs		Amino-T807	Methylene blue	SH-SY5Y	Mice	Alzheimer’s disease	[90]
MSNs	Nucleus	TAT	DOX	MCF-7	X	Cancer	[93]
MSNs		TAT	Plasmid	Hela	Mice	Cancer	[94]
MSNs		TAT	Camptothecin	HeLa	X	Cancer	[95]
MSNs		TAT	Topotecan	MDA-MB-231	Mice	Cancer	[96]
MSNs		TAT	Gemcitabine, doxorubicin	HeLa	Mice	Cancer	[97]

## References

[1] Yanagisawa T., Shimizu T., Kuroda K., Kato C. (1990). The preparation of alkyltrimethylammonium-kanemite complexes and their conversion to microporous materials. Bull. Chem. Soc. Jpn..

[2] Kresge C.T., Leonowicz M.E., Roth W.J., Vartuli J.C., Beck J.S. (1992). Ordered mesoporous molecular sieves synthesized by a liquid-crystal template mechanism. Nature.

[3] Hoffmann F., Cornelius M., Morell J., Fröba M. (2006). Silica-based mesoporous organic-inorganic hybrid materials. Angew. Chem. Int. Ed..

[4] Vallet-Regí M., Balas F., Arcos D. (2007). Mesoporous materials for drug delivery. Angew. Chem. Int. Ed..

[5] Yan Z., Meng H., Shi L., Li Z., Kang P. (2010). Synthesis of mesoporous hollow carbon hemispheres as highly efficient Pd electrocatalyst support for ethanol oxidation. Electrochem. Commun..

[6] Serrano E., Linares N., García-Martínez J., Berenguer J.R. (2013). Sol–Gel Coordination Chemistry: Building Catalysts from the Bottom-Up. ChemCatChem.

[7] Zhang Y., Zheng S., Zhu S., Ma J., Sun Z., Farid M. (2018). Evaluation of paraffin infiltrated in various porous silica matrices as shape-stabilized phase change materials for thermal energy storage. Energy Convers. Manag..

[8] Mitran R.A., Berger D., Munteanu C., Matei C. (2015). Evaluation of Different Mesoporous Silica Supports for Energy Storage in Shape-Stabilized Phase Change Materials with Dual Thermal Responses. J. Org. Chem. C.

[9] Walcarius A., Mercier L. (2010). Mesoporous organosilica adsorbents: Nanoengineered materials for removal of organic and inorganic pollutants. J. Mater. Chem..

[10] Sangvanich T., Morry J., Fox C., Ngamcherdtrakul W., Goodyear S., Castro D., Fryxell G.E., Addleman R.S., Summers A.O., Yantasee W. (2014). Novel Oral Detoxification of Mercury, Cadmium, And Lead with Thiol-Modified Nanoporous Silica. ACS Appl. Mater. Interfaces.

[11] Vallet-Regí M., Rámila A., Del Real R.P., Pérez-Pariente J. (2001). A new property of MCM-41: Drug delivery system. Chem. Mater..

[12] Jia Y., Zhang P., Sun Y., Kang Q., Xu J., Zhang C., Chai Y. (2019). Regeneration of large bone defects using mesoporous silica coated magnetic nanoparticles during distraction osteogenesis. Nanomed. Nanotechnol. Biol. Med..

[13] Gisbert-Garzarán M., Lozano D., Vallet-Regí M., Manzano M. (2017). Self-Immolative Polymers as novel pH-responsive gate keepers for drug delivery. RSC Adv..

[14] Colilla M., Izquierdo-Barba I., Vallet-Regí M. (2018). The Role of Zwitterionic Materials in the Fight against Proteins and Bacteria. Medicines.

[15] Vallet-Regí M., Lozano D., González B., Izquierdo-Barba I. (2020). Biomaterials against Bone Infection. Adv. Healthc. Mater..

[16] Wang Y., Zhao Q., Han N., Bai L., Li J., Liu J., Che E., Hu L., Zhang Q., Jiang T. (2015). Mesoporous silica nanoparticles in drug delivery and biomedical applications. Nanomed. Nanotechnol. Biol. Med..

[17] Giret S., Wong Chi Man M., Carcel C. (2015). Mesoporous-Silica-Functionalized Nanoparticles for Drug Delivery. Chem. A Eur. J..

[18] Slowing I.I., Vivero-Escoto J.L., Wu C.-W., Lin V.S. (2008). Mesoporous silica nanoparticles as controlled release drug delivery and gene transfection carriers. Adv. Drug Deliv. Rev..

[19] Paris J.L., Vallet-Regí M. (2020). Mesoporous Silica Nanoparticles for Co-Delivery of Drugs and Nucleic Acids in Oncology: A Review. Pharmaceutics.

[20] Gisbert-Garzarán M., Berkmann J.C., Giasafaki D., Lozano D., Spyrou K., Manzano M., Steriotis T., Duda G.N., Schmidt-Bleek K., Charalambopoulou G. (2020). Engineered pH-Responsive Mesoporous Carbon Nanoparticles for Drug Delivery. ACS Appl. Mater. Interfaces.

[21] Huang X., Wu S., Du X. (2016). Gated mesoporous carbon nanoparticles as drug delivery system for stimuli-responsive controlled release. Carbon N. Y..

[22] Moreira A.F., Dias D.R., Correia I.J. (2016). Stimuli-responsive mesoporous silica nanoparticles for cancer therapy: A review. Microporous. Mesoporous. Mater..

[23] Gisbert-Garzarán M., Vallet-Regí M. (2020). Influence of the Surface Functionalization on the Fate and Performance of Mesoporous Silica Nanoparticles. Nanomaterials.

[24] Argyo C., Weiss V., Bräuchle C., Bein T. (2014). Multifunctional Mesoporous Silica Nanoparticles as a Universal Platform for Drug Delivery Multifunctional Mesoporous Silica Nanoparticles as a Universal Platform for Drug Delivery. Chem. Mater..

[25] Blanco E., Shen H., Ferrari M. (2015). Principles of nanoparticle design for overcoming biological barriers to drug delivery. Nat. Biotechnol..

[26] Grodzinski P., Kircher M., Goldberg M., Gabizon A. (2019). Integrating Nanotechnology into Cancer Care. ACS Nano.

[27] Maeda H. (2015). Toward a full understanding of the EPR effect in primary and metastatic tumors as well as issues related to its heterogeneity. Adv. Drug Deliv. Rev..

[28] Etheridge M.L., Campbell S.A., Erdman A.G., Haynes C.L., Wolf S.M., McCullough J. (2013). The big picture on nanomedicine: The state of investigational and approved nanomedicine products. Nanomed. Nanotechnol. Biol. Med..

[29] Dogra P., Adolphi N.L., Wang Z., Lin Y.S., Butler K.S., Durfee P.N., Croissant J.G., Noureddine A., Coker E.N., Bearer E.L. (2018). Establishing the effects of mesoporous silica nanoparticle properties on in vivo disposition using imaging-based pharmacokinetics. Nat. Commun..

[30] Ge C., Tian J., Zhao Y., Chen C., Zhou R., Chai Z. (2015). Towards understanding of nanoparticle–protein corona. Arch. Toxicol..

[31] Natfji A.A., Ravishankar D., Osborn H.M.I., Greco F. (2017). Parameters affecting the Enhanced Permeability and Retention Effect: The need for patient selection. J. Pharm. Sci..

[32] Foroozandeh P., Aziz A.A. (2018). Insight into Cellular Uptake and Intracellular Trafficking of Nanoparticles. Nanoscale Res. Lett..

[33] Yamaguchi H., Hayama K., Sasagawa I., Okada Y., Kawase T., Tsubokawa N., Tsuchimochi M. (2018). HER2-Targeted Multifunctional Silica Nanoparticles Specifically Enhance the Radiosensitivity of HER2-Overexpressing Breast Cancer Cells. Int. J. Mol. Sci..

[34] Li L., Lu Y., Jiang C., Zhu Y., Yang X., Hu X., Lin Z., Zhang Y., Peng M., Xia H. (2018). Actively Targeted Deep Tissue Imaging and Photothermal-Chemo Therapy of Breast Cancer by Antibody-Functionalized Drug-Loaded X-Ray-Responsive Bismuth Sulfide@Mesoporous Silica Core–Shell Nanoparticles. Adv. Funct. Mater..

[35] Er Ö., Colak G.S., Ocakoglu K., Ince M., Bresolí-Obach R., Mora M., Sagristá L.M., Yurt F., Nonell S. (2018). Selective Photokilling of Human Pancreatic Cancer Cells Using Cetuximab-Targeted Mesoporous Silica Nanoparticles for Delivery of Zinc Phthalocyanine. Molecules.

[36] Zhang X., Li Y., Wei M., Liu C., Yu T., Yang J. (2019). Cetuximab-modified silica nanoparticle loaded with ICG for tumor-targeted combinational therapy of breast cancer. Drug Deliv..

[37] Dineshkumar S., Raj A., Srivastava A., Mukherjee S., Pasha S.S., Kachwal V., Fageria L., Chowdhury R., Laskar I.R. (2019). Facile Incorporation of “Aggregation-Induced Emission”-Active Conjugated Polymer into Mesoporous Silica Hollow Nanospheres: Synthesis, Characterization, Photophysical Studies, and Application in Bioimaging. ACS Appl. Mater. Interfaces.

[38] Babaei M., Abnous K., Taghdisi S.M., Amel Farzad S., Peivandi M.T., Ramezani M., Alibolandi M. (2017). Synthesis of theranostic epithelial cell adhesion molecule targeted mesoporous silica nanoparticle with gold gatekeeper for hepatocellular carcinoma. Nanomedicine.

[39] Li Y., Duo Y., Bao S., He L., Ling K., Luo J., Zhang Y., Huang H., Zhang H., Yu X. (2017). EpCAM aptamer-functionalized polydopamine-coated mesoporous silica nanoparticles loaded with DM1 for targeted therapy in colorectal cancer. Int. J. Nanomedicine.

[40] Pascual L., Cerqueira-Coutinho C., García-Fernández A., de Luis B., Bernardes E.S., Albernaz M.S., Missailidis S., Martínez-Máñez R., Santos-Oliveira R., Orzaez M. (2017). MUC1 aptamer-capped mesoporous silica nanoparticles for controlled drug delivery and radio-imaging applications. Nanomed. Nanotechnol. Biol. Med..

[41] Hanafi-Bojd M.Y., Moosavian Kalat S.A., Taghdisi S.M., Ansari L., Abnous K., Malaekeh-Nikouei B. (2018). MUC1 aptamer-conjugated mesoporous silica nanoparticles effectively target breast cancer cells. Drug Dev. Ind. Pharm..

[42] Nejabat M., Mohammadi M., Abnous K., Taghdisi S.M., Ramezani M., Alibolandi M. (2018). Fabrication of acetylated carboxymethylcellulose coated hollow mesoporous silica hybrid nanoparticles for nucleolin targeted delivery to colon adenocarcinoma. Carbohydr. Polym..

[43] Tang Y., Hu H., Zhang M.G., Song J., Nie L., Wang S., Niu G., Huang P., Lu G., Chen X. (2015). An aptamer-targeting photoresponsive drug delivery system using “off–on” graphene oxide wrapped mesoporous silica nanoparticles. Nanoscale.

[44] Alizadeh L., Alizadeh E., Zarebkohan A., Ahmadi E., Rahmati-Yamchi M., Salehi R. (2020). AS1411 aptamer-functionalized chitosan-silica nanoparticles for targeted delivery of epigallocatechin gallate to the SKOV-3 ovarian cancer cell lines. J. Nanoparticle Res..

[45] Sun X., Wang N., Yang L.-Y., Ouyang X.-K., Huang F. (2019). Folic Acid and PEI Modified Mesoporous Silica for Targeted Delivery of Curcumin. Pharmaceutics.

[46] Cheng W., Nie J., Xu L., Liang C., Peng Y., Liu G., Wang T., Mei L., Huang L., Zeng X. (2017). pH-Sensitive Delivery Vehicle Based on Folic Acid-Conjugated Polydopamine-Modified Mesoporous Silica Nanoparticles for Targeted Cancer Therapy. ACS Appl. Mater. Interfaces.

[47] Lv G., Qiu L., Liu G., Wang W., Li K., Zhao X., Lin J. (2016). pH sensitive chitosan-mesoporous silica nanoparticles for targeted delivery of a ruthenium complex with enhanced anticancer effects. Dalt. Trans..

[48] Li Z., Zhang Y., Zhang K., Wu Z., Feng N. (2018). Biotinylated-lipid bilayer coated mesoporous silica nanoparticles for improving the bioavailability and anti-leukaemia activity of Tanshinone IIA. Artif. Cells Nanomed. Biotechnol..

[49] Liu J., Zhang B., Luo Z., Ding X., Li J., Dai L., Zhou J., Zhao X., Ye J., Cai K. (2015). Enzyme responsive mesoporous silica nanoparticles for targeted tumor therapy in vitro and in vivo. Nanoscale.

[50] Villaverde G., Alfranca A., Gonzalez-Murillo Á., Melen G.J., Castillo R.R., Ramírez M., Baeza A., Vallet-Regí M. (2019). Molecular Scaffolds as Double-Targeting Agents For the Diagnosis and Treatment of Neuroblastoma. Angew. Chemie Int. Ed..

[51] Paris J.L., Villaverde G., Cabañas M.V., Manzano M., Vallet-Regí M. (2018). From proof-of-concept material to PEGylated and modularly targeted ultrasound-responsive mesoporous silica nanoparticles. J. Mater. Chem. B.

[52] Xu X., Li H., Li K., Zeng Q., Liu Y., Zeng Y., Chen D., Liang J., Chen X., Zhan Y. (2019). A photo-triggered conjugation approach for attaching RGD ligands to biodegradable mesoporous silica nanoparticles for the tumor fluorescent imaging. Nanomed. Nanotechnol. Biol. Med..

[53] Zhang P., Tang M., Huang Q., Zhao G., Huang N., Zhang X., Tan Y., Cheng Y. (2019). Combination of 3-methyladenine therapy and Asn-Gly-Arg (NGR)-modified mesoporous silica nanoparticles loaded with temozolomide for glioma therapy in vitro. Biochem. Biophys. Res. Commun..

[54] Lee J., Oh E.-T., Han Y., Kim H.G., Park H.J., Kim C. (2017). Mesoporous Silica Nanocarriers with Cyclic Peptide Gatekeeper: Specific Targeting of Aminopeptidase N and Triggered Drug Release by Stimuli-Responsive Conformational Transformation. Chem. Eur. J..

[55] Villaverde G., Gómez-Graña S., Guisasola E., García I., Hanske C., Liz-Marzán L.M., Baeza A., Vallet-Regí M. (2018). Targeted Chemo-Photothermal Therapy: A Nanomedicine Approximation to Selective Melanoma Treatment. Part. Part. Syst. Charact..

[56] Shi J., Hou S., Huang J., Wang S., Huan W., Huang C., Liu X., Jiang R., Qian W., Lu J. (2017). An MSN-PEG-IP drug delivery system and IL13Rα2 as targeted therapy for glioma. Nanoscale.

[57] Montalvo-Quiros S., Aragoneses-Cazorla G., Garcia-Alcalde L., Vallet-Regí M., González B., Luque-Garcia J.L. (2019). Cancer cell targeting and therapeutic delivery of silver nanoparticles by mesoporous silica nanocarriers: Insights into the action mechanisms using quantitative proteomics. Nanoscale.

[58] Saini K., Bandyopadhyaya R. (2020). Transferrin-Conjugated Polymer-Coated Mesoporous Silica Nanoparticles Loaded with Gemcitabine for Killing Pancreatic Cancer Cells. ACS Appl. Nano Mater..

[59] Zhou J., Li M., Lim W.Q., Luo Z., Phua S.Z.F., Huo R., Li L., Li K., Dai L., Liu J. (2018). A Transferrin-Conjugated Hollow Nanoplatform for Redox-Controlled and Targeted Chemotherapy of Tumor with Reduced Inflammatory Reactions. Theranostics.

[60] Martínez-Carmona M., Baeza A., Rodríguez-Milla M.A., García-Castro J., Vallet-Regí M. (2015). Mesoporous silica nanoparticles grafted with a light-responsive protein shell for highly cytotoxic antitumoral therapy. J. Mater. Chem. B.

[61] Martínez-Carmona M., Lozano D., Colilla M., Vallet-Regí M. (2018). Lectin-Conjugated pH-Responsive Mesoporous Silica Nanoparticles for Targeted Bone Cancer Treatment. Acta Biomater..

[62] Bhat R., García I., Aznar E., Arnaiz B., Martínez-Bisbal M.C., Liz-Marzán L.M., Penadés S., Martínez-Máñez R. (2018). Lectin-gated and glycan functionalized mesoporous silica nanocontainers for targeting cancer cells overexpressing Lewis X antigen. Nanoscale.

[63] Smith S.A., Selby L.I., Johnston A.P.R., Such G.K. (2018). The Endosomal Escape of Nanoparticles: Toward More Efficient Cellular Delivery. Bioconjug. Chem..

[64] Weiss V., Argyo C., Torrano A.A., Strobel C., Mackowiak S.A., Schmidt A., Datz S., Gatzenmeier T., Hilger I., Bräuchle C. (2016). Dendronized mesoporous silica nanoparticles provide an internal endosomal escape mechanism for successful cytosolic drug release. Microporous. Mesoporous. Mater..

[65] Chen J., Li J., Zhou J., Lin Z., Cavalieri F., Czuba-Wojnilowicz E., Hu Y., Glab A., Ju Y., Richardson J.J. (2019). Metal–Phenolic Coatings as a Platform to Trigger Endosomal Escape of Nanoparticles. ACS Nano.

[66] Chen Z., Zhu P., Zhang Y., Liu Y., He Y., Zhang L., Gao Y. (2016). Enhanced Sensitivity of Cancer Stem Cells to Chemotherapy Using Functionalized Mesoporous Silica Nanoparticles. Mol. Pharm..

[67] Sanchez-Salcedo S., Vallet-Regí M., Shahin S.A., Glackin C.A., Zink J.I. (2018). Mesoporous core-shell silica nanoparticles with anti-fouling properties for ovarian cancer therapy. Chem. Eng. J..

[68] Li S., Hong M. (2011). Protonation, Tautomerization, and Rotameric Structure of Histidine: A Comprehensive Study by Magic-Angle-Spinning Solid-State NMR. J. Am. Chem. Soc..

[69] Bilalis P., Tziveleka L.-A., Varlas S., Iatrou H. (2016). pH-Sensitive nanogates based on poly(L-histidine) for controlled drug release from mesoporous silica nanoparticles. Polym. Chem..

[70] Li Z., Zhang L., Tang C., Yin C. (2017). Co-Delivery of Doxorubicin and Survivin shRNA-Expressing Plasmid Via Microenvironment-Responsive Dendritic Mesoporous Silica Nanoparticles for Synergistic Cancer Therapy. Pharm. Res..

[71] Yadav D.K., Kumar S., Choi E.-H., Chaudhary S., Kim M.-H. (2019). Molecular dynamic simulations of oxidized skin lipid bilayer and permeability of reactive oxygen species. Sci. Rep..

[72] Hai L., Jia X., He D., Zhang A., Wang T., Cheng H., He X., Wang K. (2018). DNA-Functionalized Hollow Mesoporous Silica Nanoparticles with Dual Cargo Loading for Near-Infrared-Responsive Synergistic Chemo-Photothermal Treatment of Cancer Cells. ACS Appl. Nano Mater..

[73] Niedermayer S., Weiss V., Herrmann A., Schmidt A., Datz S., Müller K., Wagner E., Bein T., Bräuchle C. (2015). Multifunctional polymer-capped mesoporous silica nanoparticles for pH-responsive targeted drug delivery. Nanoscale.

[74] N V.R., Han H.S., Lee H., Nguyen V.Q., Jeon S., Jung D.-W., Lee J., Yi G.-R., Park J.H. (2018). ROS-responsive mesoporous silica nanoparticles for MR imaging-guided photodynamically maneuvered chemotherapy. Nanoscale.

[75] Martínez-Carmona M., Lozano D., Baeza A., Colilla M., Vallet-Regí M. (2017). A novel visible light responsive nanosystem for cancer treatment. Nanoscale.

[76] Catalán M., Olmedo I., Faúndez J., Jara J.A. (2020). Medicinal chemistry targeting mitochondria: From new vehicles and pharmacophore groups to old drugs with mitochondrial activity. Int. J. Mol. Sci..

[77] Qu Q., Ma X., Zhao Y. (2015). Targeted delivery of doxorubicin to mitochondria using mesoporous silica nanoparticle nanocarriers. Nanoscale.

[78] Cai X., Luo Y., Song Y., Liu D., Yan H., Li H., Du D., Zhu C., Lin Y. (2018). Integrating in situ formation of nanozymes with three-dimensional dendritic mesoporous silica nanospheres for hypoxia-overcoming photodynamic therapy. Nanoscale.

[79] Cheng R., Kong F., Tong L., Liu X., Xu K., Tang B. (2018). Simultaneous Detection of Mitochondrial Hydrogen Selenide and Superoxide Anion in HepG2 Cells under Hypoxic Conditions. Anal. Chem..

[80] Sun K., Gao Z., Zhang Y., Wu H., You C., Wang S., An P., Sun C., Sun B. (2018). Enhanced highly toxic reactive oxygen species levels from iron oxide core–shell mesoporous silica nanocarrier-mediated Fenton reactions for cancer therapy. J. Mater. Chem. B.

[81] Wang L., Niu X., Song Q., Jia J., Hao Y., Zheng C., Ding K., Xiao H., Liu X., Zhang Z. (2020). A two-step precise targeting nanoplatform for tumor therapy via the alkyl radicals activated by the microenvironment of organelles. J. Control. Release.

[82] Naz S., Wang M., Han Y., Hu B., Teng L., Zhou J., Zhang H., Chen J. (2019). Enzyme-responsive mesoporous silica nanoparticles for tumor cells and mitochondria multistage-targeted drug delivery. Int. J. Nanomedicine.

[83] Ahn J., Lee B., Choi Y., Jin H., Lim N.Y., Park J., Kim J.H., Bae J., Jung J.H. (2018). Non-peptidic guanidinium-functionalized silica nanoparticles as selective mitochondria-targeting drug nanocarriers. J. Mater. Chem. B.

[84] Liu J., Liang H., Li M., Luo Z., Zhang J., Guo X., Cai K. (2018). Tumor acidity activating multifunctional nanoplatform for NIR-mediated multiple enhanced photodynamic and photothermal tumor therapy. Biomaterials.

[85] Hu J.-J., Lei Q., Peng M.-Y., Zheng D.-W., Chen Y.-X., Zhang X.-Z. (2017). A positive feedback strategy for enhanced chemotherapy based on ROS-triggered self-accelerating drug release nanosystem. Biomaterials.

[86] Choi J.Y., Gupta B., Ramasamy T., Jeong J.-H., Jin S.G., Choi H.-G., Yong C.S., Kim J.O. (2018). PEGylated polyaminoacid-capped mesoporous silica nanoparticles for mitochondria-targeted delivery of celastrol in solid tumors. Colloids Surfaces B Biointerfaces.

[87] Kundu M., Chatterjee S., Ghosh N., Manna P., Das J., Sil P.C. (2020). Tumor targeted delivery of umbelliferone via a smart mesoporous silica nanoparticles controlled-release drug delivery system for increased anticancer efficiency. Mater. Sci. Eng. C.

[88] Luo G.-F., Chen W.-H., Liu Y., Lei Q., Zhuo R.-X., Zhang X.-Z. (2014). Multifunctional Enveloped Mesoporous Silica Nanoparticles for Subcellular Co-delivery of Drug and Therapeutic Peptide. Sci. Rep..

[89] Cao J., Zhang Y., Shan Y., Wang J., Liu F., Liu H., Xing G., Lei J., Zhou J. (2017). A pH-dependent Antibacterial Peptide Release Nano-system Blocks Tumor Growth in vivo without Toxicity. Sci. Rep..

[90] Chen Q., Du Y., Zhang K., Liang Z., Li J., Yu H., Ren R., Feng J., Jin Z., Li F. (2018). Tau-Targeted Multifunctional Nanocomposite for Combinational Therapy of Alzheimer’s Disease. ACS Nano.

[91] Pustylnikov S., Costabile F., Beghi S., Facciabene A. (2018). Targeting mitochondria in cancer: Current concepts and immunotherapy approaches. Transl. Res..

[92] Pan L., Shi J. (2018). Chemical Design of Nuclear-Targeting Mesoporous Silica Nanoparticles for Intra-nuclear Drug Delivery. Chin. J. Chem..

[93] Zhao J., Zhao F., Wang X., Fan X., Wu G. (2016). Secondary nuclear targeting of mesoporous silica nano-particles for cancer-specific drug delivery based on charge inversion. Oncotarget.

[94] Wu M., Meng Q., Chen Y., Du Y., Zhang L., Li Y., Zhang L., Shi J. (2015). Large-Pore Ultrasmall Mesoporous Organosilica Nanoparticles: Micelle/Precursor Co-templating Assembly and Nuclear-Targeted Gene Delivery. Adv. Mater..

[95] Wu Z.-Y., Lee C.-C., Lin H.-M. (2019). Hyaluronidase-Responsive Mesoporous Silica Nanoparticles with Dual-Imaging and Dual-Target Function. Cancers.

[96] Murugan C., Venkatesan S., Kannan S. (2017). Cancer Therapeutic Proficiency of Dual-Targeted Mesoporous Silica Nanocomposite Endorses Combination Drug Delivery. ACS Omega.

[97] Croissant J.G., Zhang D., Alsaiari S., Lu J., Deng L., Tamanoi F., AlMalik A.M., Zink J.I., Khashab N.M. (2016). Protein-gold clusters-capped mesoporous silica nanoparticles for high drug loading, autonomous gemcitabine/doxorubicin co-delivery, and in-vivo tumor imaging. J. Control. Release.

[98] Rosenholm J.M., Mamaeva V., Sahlgren C., Lindén M. (2012). Nanoparticles in targeted cancer therapy: Mesoporous silica nanoparticles entering preclinical development stage. Nanomedicine.

[99] Narayan R., Nayak Y.U., Raichur M.A., Garg S. (2018). Mesoporous Silica Nanoparticles: A Comprehensive Review on Synthesis and Recent Advances. Pharmaceutics.

[100] Phillips E., Penate-Medina O., Zanzonico P.B., Carvajal R.D., Mohan P., Ye Y., Humm J., Gönen M., Kalaigian H., Schöder H. (2014). Clinical translation of an ultrasmall inorganic optical-PET imaging nanoparticle probe. Sci. Transl. Med..

[101] Bukara K., Schueller L., Rosier J., Martens M.A., Daems T., Verheyden L., Eelen S., Van Speybroeck M., Libanati C., Martens J.A. (2016). Ordered mesoporous silica to enhance the bioavailability of poorly water-soluble drugs: Proof of concept in man. Eur. J. Pharm. Biopharm..

